# Factors motivating smoking cessation: a cross-sectional study in a lower-middle-income country

**DOI:** 10.1186/s12889-021-11477-2

**Published:** 2021-07-18

**Authors:** Russell Seth Martins, Muhammad Umer Junaid, Muhammad Sharjeel Khan, Namrah Aziz, Zoha Zahid Fazal, Mariam Umoodi, Fatima Shah, Javaid Ahmed Khan

**Affiliations:** 1 Medical College, Aga Khan University Hospital, Stadium Road, Karachi, 74800 Pakistan; 2 Altamash Institute of Dental Medicine, Block 3 Clifton, Karachi, 75500 Pakistan; 3 Darul Sehat Hospital, Gulistan-e-Johar, Karachi, 74200 Pakistan; 4 Section of Pulmonary and Critical Care Medicine, Department of Medicine, Aga Khan University Hospital, Stadium Road, Karachi, 74800 Pakistan

**Keywords:** Tobacco, Developing countries, Nicotine replacement therapy, Cigarettes, Ex-smokers

## Abstract

**Introduction:**

Only one-quarter of smokers in Pakistan attempt to quit smoking, and less than 3% are successful. In the absence of any literature from the country, this study aimed to explore factors motivating and strategies employed in successful smoking cessation attempts in Pakistan, a lower-middle-income country.

**Methods:**

A survey was carried out in Karachi, Pakistan, amongst adult (≥ 18 years) former smokers (individuals who had smoked ≥100 cigarettes in their lifetime but who had successfully quit smoking for > 1 month at the time of survey). Multivariable logistic regression, with number of quit attempts (single vs. multiple) as the dependent variable, was performed while adjusting for age, sex, monthly family income, years smoked, cigarettes/day before quitting, and having suffered from a smoking-related health problem.

**Results:**

Out of 330 former smokers, 50.3% quit successfully on their first attempt with 62.1% quitting “cold turkey”. Only 10.9% used a cessation aid (most commonly nicotine replacement therapy: 8.2%). Motivations for quitting included self-health (74.5%), promptings by one’s family (43%), and family’s health (14.8%). Other social pressures included peer-pressure to quit smoking (31.2%) and social avoidance by non-smokers (22.7%). Successful smoking cessation on one’s first attempt was associated with being married (OR: 4.47 [95% CI: 2.32–8.61]), employing an abrupt cessation mode of quitting (4.12 [2.48–6.84]), and telling oneself that one has the willpower to quit (1.68 [1.04–2.71]).

**Conclusion:**

In Pakistan, smoking cessation is motivated by concern for self-health and family’s health, family’s support, and social pressures. Our results lay a comprehensive foundation for the development of smoking-cessation interventions tailored to the population of the country.

**Implications:**

Little is known about the patterns and strategies employed by smokers who are attempting to quit smoking, especially in lower-middle-income countries like Pakistan. Likewise, there are very few smoking cessation programs designed to assist in quitting. Our study will allow for a better understanding of the culture-specific motivating factors and strategies that most contributed to successful quit attempts. Based on these results, evidence based smoking cessation interventions can be developed tailored to the socioeconomic demographic of our country and region, including smoking cessation clinics and public outreach and media campaigns highlighting key elements of successful smoking cessation.

## Introduction

Smoking, with an average of 7 million deaths per year, is currently the leading cause of preventable death in the world [1], and causes a significant burden of oral and other cancers [2]. Literature pertaining to smoking cessation has shown that around two thirds of cigarette smokers are interested in quitting, with more than 50% reporting making a quit attempt in the past year [3]. However, fewer than one third of smokers who tried to quit used proven cessation methods, with only one in 10 smokers being able to quit successfully [3]. A UK based study showed that one third of quitting attempts were not preplanned and around half of those were made without the use of any support and thus were less likely to be successful [4]. Documented and validated support-based methods, and thus by extension a plan beforehand, contribute towards the success of any quit attempt [4].

To facilitate those with the intention to quit smoking, it is imperative to identify factors motivating successful cessation in former smokers and use these to support others’ quit attempts [5]. Cessation-aid interventions that are designed according to specific motivations to quit smoking are likely to increase chances of successful cessation [6]. Factors motivating smoking cessation range from internal/individual factors (such as a smokers emotional state and willpower) and external factors (such as advice on why and how to quit from health professionals, environmental smoking restrictions, and expectations about the benefits of quitting) [7]. The importance of internal/individual factors must not be undermined, as they have been shown to affect the efficacy of smoking cessation programs [7, 8].

While there is extensive literature exploring factors motivating smoking cessation amongst populations in developed countries [9], such research is scarce from lower-middle-income countries (LMICs) such as Pakistan. Around 19.1% of Pakistan’s adult population are tobacco users, with the majority being smokers [10], and approximately 10% of deaths in Pakistan annually are attributable to smoking [11]. Apart from devastating consequences on population health, smoking also costs Pakistan approximately Rs. 192 billion (1.37 billion United States Dollars) annually due to costs associated with smoking-associated cancers, respiratory disease, and cardiovascular disease [12]. However, according to the World Health Organization (WHO) Global Adult Tobacco Survey (GATS), a much lower percentage (24.7%) of smokers in Pakistan make attempts to quit smoking, as compared to other countries (40–50%) [13, 14]. In addition, the success rates of quit attempts are also lower for smokers in Pakistan (2.6%), as compared to those reported by international literature [13]. Almost half of the smokers attempting to quit did so without assistance (49.2%) and were hence less likely to be successful, with only 9.1% making use of pharmacotherapy and 14.7% of counseling [14]. The huge gap between the number of smokers attempting to quit and those actually successful highlights the ineffectiveness or absence of adequate motivators of smoking cessation and interventions designed to motivate and support successful cessation attempts in Pakistan [15]. The GATS survey also found that almost two-thirds (63.9%) of smokers were individuals without any education and around 59.8% were not interested in quitting [14]. This calls into question the benefit of mass media campaigns for smoking cessation, particularly those using a textual medium, in a country where the majority of smokers are illiterate [14]. In addition, since most smokers in Pakistan belong from lower socioeconomic backgrounds [16], cessation aids such as pharmacotherapy and counseling may be out of the financial reach of many individuals. Lastly, cultural and religious influences on smoking practices [17] may contribute to cessation patterns that differ from those seen in Western countries.

Although the 2014 GATS survey [14] provides highly generalizable national-level data regarding the sociodemographic distribution of ex-smokers and their use of cessation aids, it did not explore factors driving cessation itself. This gap in knowledge represents a niche that invites further research. Moreover, though the GATS survey reported that 29.7% of current smokers thought of quitting because of warning labels on cigarette packages [14], the impact of other public health interventions to promote cessation was largely unexplored. Thus, this study aims to describe factors motivating successful smoking cessation attempts in Pakistan, so that these may be incorporated towards the development of smoking cessation interventions that are targeted to the population of the country. In addition, this study also aims to identify motivators and strategies that are associated with successful cessation on one’s first attempt. Lastly, our study also reports the perceived usefulness of public health interventions in motivating cessation and resisting relapse amongst ex-smokers.

## Methods

### Study setting and population

This cross-sectional survey was carried out in Karachi, Pakistan, after approval from the institutional review board at the Aga Khan University Hospital (AKUH). The target population for this survey was adult former smokers, who were defined as adult (≥ 18 years) individuals who had smoked at least 100 cigarettes in their lifetime but who had successfully quit smoking at the time of survey [18]. A quit attempt was defined as deliberately stopping smoking for > 1 week, while successful quitting was defined as having deliberately stopped smoking for > 1 month [18]. A quit attempt was categorized as unsuccessful if any smoking relapse (≥ 1 cigarette smoked) took place after a quit attempt.

### Survey characteristics

Data was collected by means of a questionnaire that was available in both English and Urdu, the national language of Pakistan. In the absence of a prior questionnaire suitable for our population, a comprehensive questionnaire was developed using elements from various sources [9, 19, 20] in close association with faculty with expertise in tobacco cessation research at the Section of Pulmonary and Critical Care Medicine at AKUH and the University of York. Content validity was assessed by calculating a content validity index (CVI) for relevance and clarity based on the ratings of three subject experts and a biostatistician. A CVI for relevance of 0.92 and for clarity of 0.89 indicated good content validity for the tool. The English questionnaire was then translated to Urdu, which is the national language of Pakistan, by an independent translator fluent in both languages and with experience in questionnaire translation. To ensure face validity, the English and Urdu versions of the survey underwent pilot testing amongst 30 respondents, and any ambiguous questions were subsequently modified as appropriate. The final survey contained the following five sections:

- *Demographics and Job Characteristics*: Age, sex, marital status, and monthly family income.
- *History of Smoking and Smoking Cessation*: Age at starting smoking, duration of smoking, number of quit attempts, cigarettes/day before cessation, time since quitting, age at cessation, difficulty of cessation (5-point Likert Scale consisting of *5 = very difficult*; *4 = difficult*; *3 = neither difficult nor easy*; *2 = easy*; and *1 = very easy*), and perceived self-efficacy in quitting (Question: “*Do you believe you have quit definitively*? Responses: *Yes/ No/Unsure*) [9].
- *Strategies Employed in Smoking Cessation*: Mode of quitting used in successful attempt (*abrupt cessation/cold turkey* vs. *gradual reduction*), use of a cessation aid (checklist of different available cessation aids) [9], strategies for self-discipline (*Yes/No* for each strategy using a checklist) [20], strategies for self-distraction from smoking (*Yes/No* for each strategy using a checklist), and positive reinforcement strategies (*Yes/No* for each strategy using a checklist) [20].
- *Factors Motivating Smoking Cessation*: Major reasons for quitting smoking (*Yes/No* for each reason using a checklist), sources of awareness regarding need to quit smoking (*Yes/No* for each source using a checklist) [19], social factors motivating cessation (*Yes/No* for each factor using a checklist), factors related to self-image (*Yes/No* for each factor using a checklist) [20], and existence of smoking-related health problems [9].
- *Usefulness of Public Health Interventions in Aiding Smoking Cessation*: The helpfulness of public health interventions in motivating cessation and resisting relapse (multiple choice responses: *not helpful at all*, *helpful to a small extent* or *helpful to a great extent*).

The survey was preceded by a consent form (available in both English and Urdu) explaining the nature and scope of the survey. In addition, preliminary screening questions based on current smoking status ensured that current smokers or those who had quit for < 1 month were not allowed to proceed with answering the survey.

### Sample size calculation

Since no published literature reports factors motivating smoking cessation in Pakistan, it was assumed that approximately 75% of former smokers will have quit for health purposes (to protect present or future health), which will be the most common reason. This figure is based on a study by Gallus et al. in 2013 that was conducted amongst 3075 former smokers in a European population [21]. The sample size required for our study was calculated using OpenEpi. Using a 95% confidence level, the minimum required sample size was determined to be 288 adult former smokers.

### Sampling technique

In order to achieve a representative sample for this study, data collection was conducted on the premises of five tertiary care hospitals (three government-owned and two privately-owned) in Karachi, including AKUH. Non-probability convenience sampling was used to recruit participants for the survey. Data collectors approached patients’ attendants (persons accompanying patients) for participation in the survey. Individuals who had presented to the hospital for reasons pertaining to their own health were not considered for inclusion. Patients’ attendants are assumed to be representative of the general population. After initially introducing the study and obtaining consent from the individual, the data collectors screened potential participants according to the inclusion criteria and exclusion criteria. If the individual were suitable for inclusion, an informed consent was obtained. A copy of the consent form was provided to the participant. Next, the data collectors verbally administered the survey in English or Urdu, according to the participant’s preference.

### Ethical considerations

To ensure privacy, the interaction of administering the survey took place in the nearest quiet location (empty room) on the hospital premises, according to the participant’s comfort. Moreover, to maintain anonymity, the questionnaire did not record respondents’ names. There were no risks, immediate benefits, or incentives for participation in the survey.

### Statistical analysis

Statistical analysis was performed using IBM SPSS version 23. Continuous data was presented using mean and standard deviation/ median (interquartile range), and compared using independent sample t-tests/Mann Whitney tests, as appropriate. Categorical data was presented using frequencies and percentages, and compared using chi-squared tests/Fischer’s Exact tests. Content validity indices (CVI) were calculated for clarity and relevance based on the ratings of three content experts and a biostatistician. Multivariable logistic regression, age, sex, monthly family income, years smoked, cigarettes/day before quitting, and having suffered from a smoking-related health problem, was performed with number of quit attempts as the dependent variable (dichotomized as *single attempt/successful on first attempt* and *multiple attempts/one or more unsuccessful attempts before a successful attempt*). A *p*-value < 0.05 was considered statistically significant for all analyses.

## Results

A total of 330 former smokers were included, with the majority male (92.7%) and aged between 18 and 30 years (43%) and 31–45 year (27.9%). Monthly family income was < Rs. 25,000 in 49.7% of respondents and > Rs. 75,000 in 18.2%. The mean age at which respondents at started smoking was 18.05 years, while the mean age at successful quitting was 31.37 years. Around half of the respondents reported having successfully quit smoking in their first attempt (50.3%), while 17.9% reported > 6 quit attempts. Most respondents reported smoking < 10 cigarettes a day (68.2%) at the time they began their successful quit attempt (Table 1).

**Table 1:** Respondent demographics and smoking history

Variable	***N*** = 330 *n* (%)/Mean ± SD
**Age Groups**	
**18–30 Years**	142 (43.0)
**31–45 Years**	92 (27.9)
**46–60 Years**	70 (21.2)
**> 60 Years**	26 (7.9)
**Sex**	
**Male**	306 (92.7)
**Female**	24 (7.3)
**Marital Status**	
**Married**	190 (57.6)
**Unmarried**	140 (42.4)
**Monthly Family Income**	
**< Rs. 25,000**	164 (49.7)
**Rs. 25,000-Rs. 50,000**	48 (14.5)
**Rs. 50,000-Rs. 75,000**	58 (17.6)
**> Rs. 75,000**	60 (18.2)
**Age at Starting (years)**	18.05 ± 3.79
**Number of Quit Attempts**	
**1 Attempts**	166 (50.3)
**2–5 Attempts**	105 (31.8)
**> 6 Attempts**	59 (17.9)
**Cigarettes/day before Quitting**	
**< 10 Cigarettes**	225 (68.2)
**≥ 10 Cigarettes**	105 (31.8)
**Age at Quitting (years)**	31.37 ± 10.77
**Duration of Smoking (years)**	13.32 ± 10.55
**Time Since Quitting (years)**	7.50 ± 8.04

The majority of respondents reported that they had abruptly stopped smoking (quit “cold turkey”; 62.1%). However, only 36 (10.9%) of respondents reported using a cessation aid during their successful quit attempt. Nicotine replacement therapy was the most common cessation aid used (*n* = 27; 8.2%). Additionally, 3 (0.9%) respondents reported using mint gums, while only 2 (0.6%) reported using pharmacological cessation therapy and 1 (0.3%) reported having attended psychotherapy/ counselling sessions for smoking cessation. Respondents also reported avoiding social company that encouraged smoking (46.4%), as well as triggers that caused an urge to smoke (28.5%). The majority of respondents believed that they had quit smoking definitively (83.9%), although the majority felt that giving up smoking was very difficult/difficult (63.9%). Respondents reported using a variety of ways to discipline or distract themselves when they felt the urge to smoke, as well as various positive reinforcement strategies to aid cessation (Table 2).

**Table 2:** Quitting strategies

Variable	*N* = 330 *n* (%)
**What mode of quitting did you primarily use when you successfully quit smoking?**	
**Abrupt Cessation/Cold Turkey**	205 (62.1)
**Gradual Reduction**	125 (37.9)
**Cessation Aid Used**	36 (10.9)
**Smoking and Self-Discipline**	
**“I told myself that it is a matter of my own choice to smoke or not”**	168 (50.9)
**“I told myself that I have the willpower with me to quit”**	128 (38.8)
**“I told myself that if I try hard enough, I can resist the urge to smoke”**	68 (20.6)
**“I made self-promises not to smoke”**	58 (17.6)
**How did you distract yourself from smoking when you felt the urge to smoke?**	
**Consciously Diverting Thoughts to Other Matters**	123 (37.3)
**Tried to Keep Hands/Fingers Occupied**	114 (34.5)
**Engaged in Work**	95 (28.8)
**Engaged in Physical Exercise**	87 (26.4)
**Engaged in Hobbies**	72 (21.8)
**Positive Reinforcement Strategies**	
**“I expected rewards from others when I successfully resisted the urge to smoke”**	78 (23.6)
**“I received rewards from others when I successfully resisted the urge to smoke”**	63 (19.1)
**“Others tried to make me feel good about myself when I resisted the urge to smoke”**	19 (5.8)
**“I rewarded myself when I successfully resisted the urge to smoke”**	16 (4.8)
**Did you avoid triggers causing the urge to smoke (such as a favorite sofa/location)?**	94 (28.5)
**Did you avoid social company that encouraged you to smoke?**	153 (46.4)
**Do you believe you have quit definitively?**	
**Yes**	277 (83.9)
**No**	13 (3.9)
**Unsure**	40 (12.1)
**How difficult was it to give up smoking?**	
**Very Difficult/Difficult**	211 (63.9)
**Neither Difficult nor Easy**	69 (20.9)
**Very Easy/Easy**	50 (15.2)
**How frequently do you think about taking up smoking again?**	
**Never**	207 (62.7)
**Sometimes**	97 (29.4)
**Often**	26 (7.9)

The most frequently reported reason for quitting smoking was to improve or protect one’s own health (74.5%), which also served to justify our earlier estimate of 75% for sample size calculation. Other common reasons included promptings by one’s family (43%), and to improve/protect the health of family members (14.8%). 38.8% of respondents reported suffering from a smoking-related health problem (38.8%). Common sources of awareness regarding the need to quit smoking included family/friends/colleagues (37.6%), doctors (24.8%) and social media/online platforms (20.6%). Certain social pressures to quit smoking, such as peer-pressure to quit smoking (31.2%) and social avoidance by non-smokers (22.7%), were also reported. Respondents also reported having felt the need to give up smoking to be content with themselves (33.3%) and having felt upset whenever they felt the urge to smoke (30.9%). The various factors that encouraged smoking cessation are shown in Table 3.

**Table 3:** Factors encouraging smoking cessation

Variable	*N* = 330 *n* (%)
**Major Reasons for Quitting Smoking**	
**To Improve/Protect Own Health**	246 (74.5)
**Family’s Promptings**	142 (43.0)
**To Improve/Protect Health of Family Member(s)**	49 (14.8)
**To Save Money**	48 (14.5)
**Doctors’ Promptings**	43 (13.0)
**Friends’ Promptings**	29 (8.8)
**Source of Awareness Regarding Need to Quit Smoking**	
**Family/Friends/Colleagues**	124 (37.6)
**Doctors**	82 (24.8)
**Social Media/Online Platforms**	68 (20.6)
**News/Magazine Articles**	43 (13.0)
**Television Advertisements/Shows**	34 (10.3)
**Social Cues/Pressures to Quit Smoking**	
**Peer-Pressure to Quit Smoking**	103 (31.2)
**Social Avoidance by Non-Smoker(s)**	75 (22.7)
**Non-Smokers Asserting Rights to Smokeless Public Spaces**	30 (9.1)
**“No-Smoking” Signs**	29 (8.8)
**Separate “Smokers” Areas in Public Spaces**	16 (4.8)
**Suffered/Suffering from a Smoking-related Health Problem**	128 (38.8)
**Smoking and Self-Image**	
**“To be content with myself, I needed to give up smoking”**	110 (33.3)
**“I would feel upset with myself whenever I felt the urge to smoke”**	102 (30.9)
**“My dependency made me feel disappointed in myself”**	98 (29.7)
**“Smoking contradicted my view of myself as caring and responsible”**	75 (22.7)

The majority of respondents felt that anti-smoking public health interventions were not helpful at all. Consumer warnings on cigarette packs (4.5%), increased prices/taxes on cigarettes (4.5%), and smoke-free public recreational places (4.2%) were most commonly reported to be helpful to a great extent in motivating cessation. Similarly, increased prices/taxes on cigarettes (4.8%) and consumer warnings on cigarette packs (4.2%) were most frequently reported to be help to a great extent in resisting relapse (Table 4).

**Table 4:** Usefulness of Public Health interventions in Motivating Smoking Cessation and Resisting Relapse

To what extent were the following Public Health Interventions helpful in motivating cessation and resisting relapse? ^a^	Motivating Cessation *N* = 330 *n* (%)	Resisting Relapse *N* = 330 *n* (%)
**Government Mass Media Anti-Smoking Campaigns**		
**Not Helpful At All**	249 (75.5)	264 (80.0)
**Helpful to a Small Extent**	80 (24.2)	65 (19.7)
**Private Sector Mass Media Anti-Smoking Campaigns**		
**Not Helpful At All**	254 (77.0)	251 (76.1)
**Helpful to a Small Extent**	76 (23.0)	79 (23.9)
**Anti-Smoking Advertisements**		
**Not Helpful At All**	249 (75.5)	248 (75.2)
**Helpful to a Small Extent**	80 (24.2)	78 (23.6)
**Decreased Cigarette/Tobacco Company Advertisements**		
**Not Helpful At All**	247 (74.8)	254 (76.4)
**Helpful to a Small Extent**	83 (25.2)	76 (23.0)
**Consumer Warnings on Cigarette Packs**		
**Not Helpful At All**	228 (69.1)	229 (69.4)
**Helpful to a Small Extent**	87 (26.4)	87 (26.4)
**Health Warnings Preceding/During Films**		
**Not Helpful At All**	262 (79.4)	265 (80.3)
**Helpful to a Small Extent**	60 (18.2)	57 (17.3)
**Increasing Prices/Taxes on Cigarettes**		
**Not Helpful At All**	221 (67.0)	221 (67.0)
**Helpful to a Small Extent**	94 (28.5)	93 (28.2)
**Smoke-Free Public Recreational Spaces**		
**Not Helpful At All**	250 (75.8)	252 (76.4)
**Helpful to a Small Extent**	66 (20.0)	78 (23.6)
**Smoke-Free Workplaces**		
**Not Helpful At All**	237 (71.8)	234 (70.9)
**Helpful to a Small Extent**	92 (28.2)	96 (29.1)
**No-Smoking Signs in Public Places**		
**Not Helpful At All**	265 (80.3)	258 (78.2)
**Helpful to a Small Extent**	65 (19.7)	72 (21.8)

^a^ Responses in “Helpful to a Great Extent” not shown (< 5% of responses)

On multivariable logistic regression (Table 5), successful smoking cessation on one’s first attempt was associated with being married (OR: 4.47 [95% CI: 2.32–8.61]), employing an abrupt cessation mode of quitting (4.12 [2.48–6.84]), the belief that smoking contradicted ones view of being caring and responsible (2.69 [1.52–4.77]), telling oneself that one has the willpower to quit (1.68 [1.04–2.71]), telling oneself that one can resist the urge to smoke if one tries hard enough (2.65 [1.45–4.84]), and consciously diverting ones thoughts to other matters when faced by the urge to smoke (2.22 [1.35–3.65]). Use of a cessation aid (0.20 [0.08–0.48]) and reporting family’s promptings as a major reason for quitting smoking (0.51 [0.32–0.82]) were inversely associated with successful cessation on first attempt (i.e., associated with one or more failed quit attempts before a successful attempt).

**Table 5:** Logistic Regression with Single Attempt at Quitting/ Successful on First Attempt

Variable	Successful on First Attempt		Successful on First Attempt	
	Crude OR [95% CI]	P-Value	Adjusted ^a^ OR [95% CI]	*P*-Value
**Age Groups**				
**18–30 Years**	Reference		Reference	
**31–45 Years**	**2.54 (1.48–4.37)**	**0.001**	0.39 (0.12–1.26)	0.115
**46–60 Years**	1.67 (0.94–2.98)	0.081	1.23 (0.44–3.44)	0.699
**> 60 Years**	1.74 (0.75–4.03)	0.197	0.78 (0.28–2.16)	0.628
**Sex**				
**Male**	1.76 (0.75–4.14)	0.197	1.28 (0.52–3.18)	0.593
**Female**	Reference		Reference	
**Monthly Family Income**				
**< Rs. 25,000**	Reference		Reference	
**Rs. 25,000-Rs. 50,000**	0.73 (0.38–1.40)	0.347	0.62 (0.32–1.23)	0.172
**Rs. 50,000-Rs. 75,000**	1.03 (0.56–1.87)	0.936	0.75 (0.37–1.53)	0.426
**> Rs. 75,000**	1.54 (0.84–2.80)	0.161	1.35 (0.69–2.66)	0.384
**Cigarettes/day before Quitting**				
**< 10 Cigarettes**	Reference		Reference	
**≥ 10 Cigarettes**	0.90 (0.57–1.44)	0.667	0.753 (0.455–1.246)	0.270
**Duration of Smoking (years)**	1.01 (0.99–1.03)	0.310	0.98 (0.95–1.02)	0.336
**Suffered/Suffering from a Smoking-related Health Problem**	1.20 (0.77–1.87)	0.415	0.92 (0.53–1.61)	0.777
**Marital Status**				
**Married**	**2.82 [1.89–4.44]**	**< 0.001**	**4.47 [2.32–8.61]**	**0.001**
**Unmarried**	Reference		Reference	–
**Mode of Quitting for Successful Attempt**				
**Abrupt Cessation/Cold Turkey**	**3.81 [2.37–6.12]**	**< 0.001**	**4.12 [2.48–6.84]**	**< 0.001**
**Gradual Reduction**	Reference		Reference	**–**
**Cessation Aid Used**	**0.26 [0.16–0.42]**	**< 0.001**	**0.20 [0.08–0.48]**	**< 0.001**
**Major Reasons for Quitting Smoking**				
**To Improve/Protect Own Health**	1.23 [0.75–2.02]	0.411	1.17 [0.66–2.07]	0.584
**Family’s Promptings**	**0.57 [0.36–0.88]**	**0.011**	**0.51 [0.32–0.82]**	**0.005**
**To Improve/Protect Health of Family Member**	1.68 [0.91–3.13]	0.100	1.53 [0.69–3.39]	0.291
**To Save Money**	0.81 [0.44–1.50]	0.503	0.90 [0.45–1.79]	0.766
**Doctors’ Promptings**	0.94 [0.49–1.78]	0.837	0.70 [0.34–1.43]	0.325
**Friends’ Promptings**	0.49 [0.22–1.09]	0.079	0.45 [0.20–1.04]	0.063
**Smoking and Self-Image**				
**“To be content with myself, I needed to give up smoking”**	1.22 [0.77–1.93]	0.392	1.35 [0.84–2.19]	0.217
**“I would feel upset with myself whenever I felt the urge to smoke”**	0.88 [0.55–1.40]	0.582	0.82 [0.49–1.37]	0.451
**“My dependency made me feel disappointed in myself”**	0.88 [0.55–1.40]	0.580	0.85 [0.52–1.40]	0.526
**“Smoking contradicted my view of myself as caring and responsible”**	**2.59 [1.50–4.46]**	**0.001**	**2.69 [1.52–4.77]**	**0.001**
**Smoking and Self-Discipline**				
**“I told myself that it is a matter of my own choice to smoke or not”**	1.44 [0.93–2.22]	0.099	1.57 [0.99–2.48]	0.053
**“I told myself that I have the willpower within me to quit”**	**1.64 [1.05–2.56]**	**0.030**	**1.68 [1.04–2.71]**	**0.034**
**“I told myself that if I try hard enough, I can resist the urge to smoke”**	**2.47 [1.41–4.35]**	**0.002**	**2.65 [1.45–4.84]**	**0.002**
**“I made self-promises not to smoke”**	0.91 [0.51–1.60]	0.734	1.08 [0.59–2.00]	0.800
**How did you distract yourself when you felt the urge to smoke?**				
**Consciously diverting thoughts to other matters**	**2.10 [1.33–3.32]**	**0.001**	**2.22 [1.35–3.65]**	**0.002**
**Tried to Keep Hands/Fingers Occupied**	0.75 [0.48–1.18]	0.216	0.69 [0.43–1.11]	0.127
**Engaged in Work**	1.36 [0.84–2.20]	0.206	1.20 [0.73–1.99]	0.472
**Engaged in Physical Exercise**	0.61 [0.37–1.01]	0.053	0.65 [0.38–1.09]	0.103
**Engaged in Hobbies**	0.99 [0.58–1.66]	0.954	1.07 [0.62–1.87]	0.803

^a^ adjusted for age, sex, monthly family income, years smoked, cigarettes/day before quitting, suffered from a smoking-related health problem

## Discussion

This study was conducted to explore factors associated with successful smoking cessation in former smokers in Pakistan, a lower-middle-income country (LMIC) in South Asia. Our study identified personal health, promptings from one’s family, and one’s family’s health, as the most important motivating factors. Social pressures to quit smoking included peer-pressure to quit and social avoidance by non-smokers. Lastly, successful cessation on one’s first quit attempt was associated with being married, quitting cold turkey, having a negative self-image of oneself due to smoking, and having strong willpower to quit.

The commonest reasons for quitting smoking were to improve/protect own health (74.5%), family’s promptings (43%), to improve/protect the health of family members (14.8%), and to save money (14.5%). Respondents reported receiving awareness regarding the need to quit smoking most commonly from their family, friends, and colleagues (37.6%). Moreover, social pressures, such as peer-pressure to quit smoking (31.2%), social avoidance by non-smokers (22.7%), and non-smokers asserting rights to smokeless public spaces (9.1%), were also major deterrents. Studies from the United States, Poland and France have demonstrated similar results, with health concerns, discouragement of smoking at home, and the high cost of cigarettes being important deterrents [22–24]. In addition, social pressure, such as having a smoke-free social network that pressurizes towards cessation, has also been found to be a strong motivator of cessation across different populations [23–25]. It is interesting that promptings by doctors were reported as being a reason for quitting by only 13% of respondents, and only one quarter (24.8%) of respondents received cessation-related awareness from their doctors. A study from the United Kingdom revealed that most patients were skeptical about doctors smoking cessation advice, which was often generic and of a preaching nature, and suggested that doctors should practice a more personalized approach to cessation counseling [26].

Around half (50.3%) of the respondents in our study reported quitting successfully on their first attempt, while the remaining reported needing 2–5 attempts (31.8%) and > 6 attempts (17.9%). These findings are in great contrast with what is usually suggested by smoking cessation programs. These vary from 8 to 14 attempts, as suggested by The American Cancer Society, the Australian Cancer Council, and the Centers for Disease Control [27–29]. However, there is some literature that aligns with our findings, as it has been suggested that though the number of quit attempts may be quite high on average, between 40 and 52% may be successful on their first serious attempt [30, 31].

On multivariable regression, successful cessation on first attempt was associated with being married, quitting cold turkey, having a negative self-image on oneself because of being a smoker, telling oneself they have the willpower to resist the urge to smoke and quit definitively, and consciously diverting one’s thoughts to distract oneself from smoking. While the concept of willpower has been debated for a long time for its actual contribution to smoking cessation [32], it has been demonstrated to be an important factor in Pakistan previously [13]. Moreover, personal willpower is an essential feature of the “5A’s” model in “Treating Tobacco Use and Dependence” [33], of which the first three A’s build towards willingness to quit and the last two A’s facilitate those willing to quit to take the final decision to quit. This concept of personal willpower being an important factor in single-attempt cessation is strengthened by how family’s promptings as a major reason for cessation was negatively associated with single-attempt cessation in our study. This suggests how personal motivation that arises from within the individual is more likely to lead to successful cessation than when it arises externally. Additionally, quitting cold turkey has been recommended as more successful in smoking cessation, as compared to gradually tapering off cigarette use [34]. Interestingly in our study, use of a smoking cessation aid was negatively associated with quitting on the first attempt, a finding corroborated by a survey by Manis et al. in Switzerland [35]. With regards to self-image, while having a negative self-image due to one’s addiction may cause distress to the smoker [36], it can also function as a powerful motivator to quit smoking as it negates the perceived benefits of smoking [37]. Lastly, being with a spouse or partner who is a non-smoker, a former smoker, or who encourages and motivates quitting, is associated with a greater likelihood of success on cessation attempts [38–40].

Self-distraction by consciously diverting one’s thoughts to other matters (37.3%), trying to keep one’s hands and fingers occupied (34.5%), and engaging in work (28.8%), were useful strategies reportedly used by respondents. Moreover, consciously diverting one’s thoughts to other matters was significantly associated with single-attempt cessation on multivariable regression. These are encouraging findings, as they are simple yet effective. More technological methods of distraction, such as mobile phone applications and games [41, 42], that have been piloted in the setting of developed countries may not be feasible for a resource-constrained like Pakistan. In addition, positive reinforcement strategies, such as expecting rewards (23.6%) and receiving rewards (19.1%) from others for resisting the urge to smoke, were also employed by respondents. Rewards and incentives, often monetary, are helpful in motivating smoking cessation, especially when individualized [43, 44].

Lastly, none of the public health interventions mentioned in our survey were perceived by respondents as particularly useful for helping smoking cessation or resisting relapse, with less than 5% of respondents rating any intervention as helpful to a great extent. This is in direct contrast with studies from developed countries, such as the United States [45, 46], and may be explained by several reasons. Firstly, interventions such as government or private sector mass media anti-smoking campaigns, anti-smoking advertisements, and health warnings preceding/during films, may not effectively be effective amongst those of lower socioeconomic and less educated backgrounds. Secondly, although Pakistan subscribes to the MPOWER model of tobacco control outlined by the World Health Organization [47], it is possible that these interventions are not practically implemented in an optimal manner. Thirdly, since our results highlight how former smokers predominantly attribute the success of their cessation to personal factors, such as willpower, self-discipline, and distraction strategies, they are perhaps unable or hesitant to acknowledge the potentially subconscious impact of external motivators. Nevertheless, further studies are required to determine the efficacy of such large-scale public health interventions in the setting of a LMIC like Pakistan, in terms of both improving cessation and cost-effectiveness.

Despite the major burden of tobacco consumption in the country, Pakistan lacks any major smoking cessation programs or clinics facilitating rehabilitation, which along with the low cost and easy availability of tobacco, can prove the difficult task of quitting even more challenging [13]. The results of our study provide a comprehensive and unique understanding of the factors that motivate smoking cessation in Pakistan. However, despite the varied distribution of socio-demographic characteristics achieved by targeting five different settings for data collection, the convenience sampling methodology used may limit the degree of generalizability of our findings to other populations in Pakistan. Nevertheless, our findings can help guide the development of evidence-based programs for smoking cessation in Pakistan and lay the foundation for similar larger-scale national research. Other potential limitations include the self-reported nature of our data as well as the possibility of social desirability bias. Future research must investigate motivators, strategies, and patterns specific to sex, age, socioeconomic status, education level, and other demographics.

## Conclusion

Major motivations for smoking cessation in a Pakistani population include to protect the health of oneself or family members, and due to promptings from family members. Self-discipline, personal willpower, distraction strategies, and positive reinforcement play an important role in a population where smoking cessation aids may be inaccessible to many. Moreover, peer-pressure to quit and social exclusion also motivate smokers towards quitting, as does the negative self-image one associates with themselves because of their addiction to smoking. Lastly, most public health interventions, such as mass media campaigns and anti-tobacco advertisements, were not perceived as being helpful for motivating cessation.

## Data Availability

Saw the data is available from the authors on reasonable request, and is not available to be shared publicly due to constraints of the institutional review board at the Aga Khan University.

## References

[1] Organization WH. WHO report on the global tobacco epidemic, 2017: monitoring tobacco use and prevention policies: World Health Organization; 2017

[2] Jiang X, Wu J, Wang J, Huang R (2019). Tobacco and oral squamous cell carcinoma: A review of carcinogenic pathways. Tob Induc Dis..

[3] Babb S (2017). Quitting smoking among adults—United States, 2000–2015. MMWR Morb Mortal Wkly Rep.

[4] Murray RL (2009). Investigating and increasing smokers’ use of effective cessation support: University of Nottingham.

[5] Diviak KR (1999). Smoking cessation: the effect of a brief motivation enhancement intervention.

[6] Williams RJ (2011). Assessing how to increase smokers' motivation to quit: [Honolulu]:[University of Hawaii at Manoa],[may 2011].

[7] Reichmann SK (1992). The role of motivation in the process of smoking cessation.

[8] Tatarkiewicz IA (2009). Factors influencing the effectiveness of smoking cessation messages.

[9] Sieminska A, Buczkowski K, Jassem E, Lewandowska K, Ucinska R, Chelminska M (2008). Patterns of motivations and ways of quitting smoking among polish smokers: a questionnaire study. BMC Public Health.

[10] WHO. Tobacco control in Pakistan. 2015; Available from: https://www.who.int/tobacco/about/partners/bloomberg/pak/en/.

[11] Evaluation) IIfHMa. Global Burden of Disease (GBD). University of Washington; 2019; Available from: https://vizhub.healthdata.org/gbd-compare/.

[12] Saqib MAN, Malik A, Rafique I, Raza FA, Ullah O, Sajjad SF, et al. Economic burden of smoking attributed illnesses in Pakistan. 2020.

[13] Shaheen K, Oyebode O, Masud H (2018). Experiences of young smokers in quitting smoking in twin cities of Pakistan: a phenomenological study. BMC Public Health.

[14] WHO (2014). Global Adult Tobacco Survey Pakistan Data.

[15] Abdullah A, Husten C (2004). Promotion of smoking cessation in developing countries: a framework for urgent public health interventions. Thorax..

[16] Gilani SI, Leon DA. Prevalence and sociodemographic determinants of tobacco use among adults in Pakistan: findings of a nationwide survey conducted in 2012. Popul Health Metr. 2013;11(1):16. 10.1186/1478-7954-11-16 PMC3850735 24004968

[17] Bush J, White M, Kai J, Rankin J, Bhopal R. Understanding influences on smoking in Bangladeshi and Pakistani adults: community based, qualitative study. 2003;326(7396):962. 10.1136/bmj.326.7396.962 PMC153853 12727770

[18] CDC. NHIS - Adult Tobacco Use - Glossary. 2017; Available from: https://www.cdc.gov/nchs/nhis/tobacco/tobacco_glossary.htm.

[19] Group GYTS. Global Youth Tobacco Survey (GYTS): Core Questionnaire With Optional Questions, Version 1.0. Centers for Disease Control and Prevention Atlanta; 2012.

[20] Prochaska JO, Velicer WF, DiClemente CC, Fava J (1988). Measuring processes of change: applications to the cessation of smoking. J Consult Clin Psychol.

[21] Gallus S, Muttarak R, Franchi M, Pacifici R, Colombo P, Boffetta P, Leon ME, la Vecchia C (2013). Why do smokers quit?. EurJ Cancer Prevent.

[22] Buczkowski K, Marcinowicz L, Czachowski S, Piszczek E (2014). Motivations toward smoking cessation, reasons for relapse, and modes of quitting: results from a qualitative study among former and current smokers. Patient Prefer Adherence.

[23] Baha M, Le Faou AL (2010). Smokers' reasons for quitting in an anti-smoking social context. Public Health.

[24] Duncan CL, Cummings SR, Hudes ES, Zahnd E, Coates TJ (1992). Quitting smoking: reasons for quitting and predictors of cessation among medical patients. J Gen Intern Med.

[25] See JHJ, Yong TH, Poh SLK, Lum YC (2019). Smoker motivations and predictors of smoking cessation: lessons from an inpatient smoking cessation programme. Singap Med J.

[26] Butler CC, Pill R, Stott NC (1998). Qualitative study of patients' perceptions of doctors' advice to quit smoking: implications for opportunistic health promotion. BMJ..

[27] American CS (2015). Guide to Quitting Smoking. J Oklahoma State Med Assoc.

[28] Ellerman A, Ford C, Stillman S. Smoking cessation: 7.7 Personal factors associated with quitting. Tobacco in Australia: facts and. 2008(3rd).

[29] Health UDo, Services H. Women and smoking: a report of the surgeon general. Washington: Public Health Service, Office of the Surgeon General. 2001.

[30] Fiore MC, Jaén CR, Baker TB, Bailey WC, Benowitz NL, Curry SJ (2008). Treating tobacco use and dependence: 2008 update.

[31] Ashley M, Cohen J, Bull S, Poland B, Gao J, Stockton L (1997). Smoking in Ontario: analysis of data from the “Q&Q” study. Prepared for Health Canada and the Ontario Ministry of Health.

[32] White C, Oliffe JL, Bottorff JL. Tobacco and the invention of quitting: a history of gender, excess and will-power. Sociol Health Illn. 2013;35(5):778–92. Epub 04/21. 10.1111/j.1467-9566.2012.01529.x PMC3757310 23600564

[33] A clinical practice guideline for treating tobacco use and dependence: A US Public Health Service report. The Tobacco Use and Dependence Clinical Practice Guideline Panel, Staff, and Consortium Representatives. Jama. 2000;283(24):3244–54. Epub 2000/06/24. 10866874

[34] Smith DK, Miller DE, Mounsey A (2017). PURLs: "cold Turkey" works best for smoking cessation. J Fam Pract.

[35] Manis M, Tamm M, Stolz D (2018). Unaided smoking cessation in healthy employees. Respiration..

[36] Zuzelo PR (2017). Smokers' guilt and shame: reactions to smoking and to Providers' cessation efforts. Holist Nurs Pract.

[37] Klein H, Sterk CE, Elifson KW. Smoke and Mirrors: The Perceived Benefits of Continued Tobacco use Among Current Smokers. Health Psychol Res. 2014;2(2):1519-. 10.4081/hpr.2014.1519 PMC4768546 26973934

[38] Margolis R, Wright L. Better Off Alone Than With a Smoker: The Influence of Partner's Smoking Behavior in Later Life. J Gerontol B Psychol Sci Soc Sci. 2016;71(4):687–97. Epub 02/17. 10.1093/geronb/gbu220 PMC4903033 25693998

[39] Coppotelli HC, Orleans CT (1985). Partner support and other determinants of smoking cessation maintenance among women. J Consult Clin Psychol.

[40] Roski J, Schmid LA, Lando HA (1996). Long-term associations of helpful and harmful spousal behaviors with smoking cessation. Addict Behav.

[41] DeLaughter KL, Sadasivam RS, Kamberi A, English TM, Seward GL, Chan SW, et al. Crave-Out: A Distraction/Motivation Mobile Game to Assist in Smoking Cessation. JMIR Serious Games. 2016;4(1):e3-e. 10.2196/games.4566 PMC4901191 27229772

[42] Ploderer B, Smith W, Pearce J, Borland R. A mobile app offering distractions and tips to cope with cigarette craving: a qualitative study. JMIR Mhealth Uhealth. 2014;2(2):e23-e. 10.2196/mhealth.3209 PMC4114415 25099632

[43] Bruijnzeel AW (2017). Reward processing and smoking. Nicotine Tob Res.

[44] Sigmon SC, Patrick ME. The use of financial incentives in promoting smoking cessation. Preventive medicine. 2012;55 Suppl(Suppl):S24–32. Epub 2012/04/25. 10.1016/j.ypmed.2012.04.007 PMC3411852 22525802

[45] Emery S, Kim Y, Choi YK, Szczypka G, Wakefield M, Chaloupka FJ. The effects of smoking-related television advertising on smoking and intentions to quit among adults in the United States: 1999–2007. Am J Public Health. 2012;102(4):751–7. Epub 02/16. 10.2105/AJPH.2011.300443 PMC3489369 22397350

[46] Chaloupka FJ, Yurekli A, Fong GT (2012). Tobacco taxes as a tobacco control strategy. Tob Control.

[47] Organization WH (2008). MPOWER: a policy package to reverse the tobacco epidemic.

